# A Two-Phase Machine Learning Framework for Context-Aware Service Selection to Empower People with Disabilities

**DOI:** 10.3390/s22145142

**Published:** 2022-07-08

**Authors:** Abdallah Namoun, Adnan Ahmed Abi Sen, Ali Tufail, Abdullah Alshanqiti, Waqas Nawaz, Oussama BenRhouma

**Affiliations:** 1 Faculty of Computer and Information Systems, Islamic University of Madinah, Madinah 42351, Saudi Arabia; adnanmnm@iu.edu.sa (A.A.A.S.); amma@iu.edu.sa (A.A.); wnawaz@iu.edu.sa (W.N.); oussama.benrhouma@iu.edu.sa (O.B.); 2 School of Digital Science, Universiti Brunei Darussalam, Tungku Link, Gadong BE1410, Brunei; ali.tufail@ubd.edu.bn

**Keywords:** service selection, disabled people, web services, quality of service, QoS, accessibility, assistive technologies, universal design, machine learning, ontologies

## Abstract

The use of software and IoT services is increasing significantly among people with special needs, who constitute 15% of the world’s population. However, selecting appropriate services to create a composite assistive service based on the evolving needs and context of disabled user groups remains a challenging research endeavor. Our research applies a scenario-based design technique to contribute (1) an inclusive disability ontology for assistive service selection, (2) semi-synthetic generated disability service datasets, and (3) a machine learning (ML) framework to choose services adaptively to suit the dynamic requirements of people with special needs. The ML-based selection framework is applied in two complementary phases. In the first phase, all available atomic tasks are assessed to determine their appropriateness to the user goal and profiles, whereas in the subsequent phase, the list of service providers is narrowed by matching their quality-of-service factors against the context and characteristics of the disabled person. Our methodology is centered around a myriad of user characteristics, including their disability profile, preferences, environment, and available IT resources. To this end, we extended the widely used QWS V2.0 and WS-DREAM web services datasets with a fusion of selected accessibility features. To ascertain the validity of our approach, we compared its performance against common multi-criteria decision making (MCDM) models, namely AHP, SAW, PROMETHEE, and TOPSIS. The findings demonstrate superior service selection accuracy in contrast to the other methods while ensuring accessibility requirements are satisfied.

## 1. Introduction

One in seven people in the world has some type of disability. Moreover, approximately 466 million persons suffer from hearing loss, and 75 million persons require access to a wheelchair [1]. Unfortunately, these figures are expected to increase as people age over time. Disabilities are diverse in nature, ranging from physical (e.g., difficulty walking) and cognitive (e.g., Alzheimer’s) to sensory (e.g., color blindness or deafness) impairments. Although non-disabled and disabled people share the same needs, available technology services, regardless of their genre (e.g., web services, mobile services, IoT services, cloud services, etc.) and domain of application (health, education, entertainment, etc.), remain insufficient and largely inaccessible by people with disabilities [2]. Although modern computing frameworks (e.g., IoT, pervasive computing, ambient intelligence) and assistive technologies are acknowledged to assist disabled people lead an independent lives (e.g., smart homes), the development of these technologies is still undervalued and inadequate [3]. 

One way to augment the number of assistive services offered to people with disabilities is to capitalize on the computing paradigms that support modularity and software reuse, such as service-oriented architecture (SOA) and microservices. SOA coupled with other computing paradigms, e.g., cloud computing and the Internet of Things, has empowered companies and organizations to fuse heterogeneous services to attain competitive advantages (i.e., through reusability and interoperability) and to satisfy the evolving business requirements of users [4,5,6]. Notably, service selection is a crucial phase to produce fulfilling and efficient service compositions. However, choosing the right services from a large sample of candidate services to accomplish user goals and satisfy preferences remains a complex research challenge [7,8]. The selection of services is complicated by several factors, such as the interoperability of services, the evolving nature of user needs, and the quality factors of available services [9]. The goal of the present research is to develop a new ML framework for selecting services to facilitate the creation of assistive services for disabled people. 

Service selection can be achieved by applying various methods [10,11,12]. The selection process is complex because it needs to satisfy several constraints and may change dynamically based on the task at hand. Three main categories of service selection stand out in the literature, namely decision making, fuzzy, and metaheuristic approaches [12]. For instance, multi-criteria decision-making (MCDM) methods [13] have been proposed and researched extensively to deal with the service selection dilemma [14,15]. Moreover, a review of previous research on service selection [16,17,18] reveals that intelligent approaches for choosing accessible services are limited, to say the least. In our methodology, we first determine the assistive tasks that support the activities of the disabled users through a multi-task classification approach and then propose the best service providers for each relevant task using a dynamic regressor model. 

The specific contributions of this research can be summarized in five distinct points, as follows.

1. A two-phase machine learning framework to optimize the selection of accessible services while satisfying various disability needs and constraints;
2. Extended datasets of accessible web services containing relevant disability aspects (e.g., types of disability supported, interaction modalities, physical environment, etc.). The accessibility datasets are deposited online for reuse by other researchers and practitioners in the field;
3. A succinct ontology of accessibility aspects to empower assistive service selection;
4. A practical solution was devised by following a scenario-based design approach. The approach was effective in motivating and guiding various activities of our research;
5. Multi-criteria consideration of the context of use, user capabilities, and preferences during the selection process. We devised an approach to evaluate and rank services based on a wealth of service quality properties.

## 2. Background

In this section, we introduce the concept of service selection and report on recent research related to service selection. Despite their scarcity, we also reviewed studies on service selection tailored for disabled users. We close this section with a comparative table of the main service selection approaches (Table 1).

### 2.1. Service Selection

The service selection process identifies one or more services from a relevant pool of services based on diverse user-provided criteria/constraints to fulfill the desired objective or complex operation [19]. In the context of service composition, services are selected during design, deployment, or runtime [20]. There are various approaches for service selection, which can be grouped into centralized, decentralized, and hybrid approaches [10].

Service selection can be achieved in a myriad of environments, such as IoT [10,11], cloud infrastructure [12], and ambient and elderly assisted living [21,22]. The authors of [10] presented a study to compare and analyze service selection strategies classified as centralized, decentralized, and hybrid. Centralized approaches rely on various factors during service selection, such as minimizing energy consumption of IoT devices in a dynamic service selection approach; achieving improved accuracy using service invocation time, location, and frequency; and a highly dynamic service selection strategy based on fuzzy logic and re-enforcement learning in an uncertain environment to achieve ease of access, use, and low-cost deployment. Decentralized approaches deal with heterogeneity, scalability, and interoperability due to an excessive number of IoT devices or associated services. One approach considers rating values or reputations associated with each service based on user preferences towards selecting a service. On the other hand, a node can forecast the ratings of neighboring nodes using a graph-based collaborative filtering approach with an inherent limitation, i.e., prediction is difficult if no rating is available. Bio-inspired decentralized approaches are prevalent in IoT for microservice selection problems. Hybrid approaches combine the merits of centralized and decentralized strategies to provide improved service selection. For instance, the authors of a study formulated a service selection strategy for smart objects using the shortest path approach in a MapReduce environment to achieve scalability and efficiency. In another study, a four-step preference-based IoT dynamic service selection approach was discussed, whereby user preferences are acquired, services are matched with the user profile, and results are communicated to the user.

In [11], the authors proposed a classification system to review and analyze various service selection algorithms within the IoT context. Service selection algorithms are grouped based on a process time phase (i.e., static at design time or dynamic at runtime), behavioral workflow (i.e., service orchestration—centralization and service choreography—decentralized), and types of optimization objectives (i.e., single, multiple, and many objectives). Many techniques in this study used either heuristic or metaheuristic approaches for service selection, whereby given QoS parameters, e.g., cost, execution time, reliability, availability, energy consumption, throughput, packet delivery rate, etc., are satisfied. Service selection is achieved in one of the three layers, namely the sensor layer, network layer, or application layer. Service selection in the sensor layer is accomplished by filtering and selecting appropriate sensors to solve the problem. For instance, sensors are modeled in a graph and applied with various shortest path algorithms to find a set of services; another approach uses a colocation-based strategy to find the correct sensors for services in a smart home scenario. In the network layer, service selection is influenced by communication energy cost and sensors energy consumption, whereas services in the application layer are selected based on objective and subjective information provided by the service provider and consumer. Similarly, the authors of [15] regarded the service selection problem as an NP-hard problem and presented a comprehensive study on service selection techniques in the cloud environment, wherein multiple decision-making, metaheuristic, and fuzzy logic approaches were analyzed on the basis of different qualitative parameters, including efficiency, availability, time, cost, scalability, and reliability.

### 2.2. Service Selection for People with Disabilities

The service selection process is non-trivial when the context and requirements are difficult to determine, e.g., people with disabilities. Therefore, researchers have devised various approaches for people with disabilities and for different environments, e.g., ambient assisted living [21], older adults [22], IoT [23,24], handicapped or elderly people [25], IoT and smart cities [26,27], and virtual education [28]. In a recent study [21], the authors presented an autonomous solution for service selection to assist elderly people in emergencies. One or more services are selected automatically using predetermined semantic rules and statistical algorithms applied to the contextual information related to user needs or preferences. All the required information is accessible through three ontologies, namely user, sensor, and service ontology. User ontology provides user profile information, and sensor ontology helps acquire data from sensors deployed in the environment to assess the context, whereas service ontology defines service specification and usage statistics. The matchmaking algorithm determines appropriate services based on the relevance of a service to a particular situation. The authors of [22] proposed a system whereby services and service providers are best matched with the customers’ (elderly) care needs. The selection process involves choosing a set of services from a pool of services that cover some care needs of the customers as potential solution fragments. These fragments are then ranked according to user needs (complying with the user profile information), and the top fragments are selected to form an integrated solution. Similarly, service selection was achieved in a robotics environment, as discussed in [25], whereby the demands of elderly or handicapped people are analyzed to determine the appropriate robotic service to fulfill user requirements. The authors did not reveal details of their robotic service selection algorithm in their study.

In the context of IoT and restful web services, the authors of [23] proposed a service composition strategy based on semantic ontology created by a domain expert. User-requested goals are fulfilled either in a semi-automated or fully automatic manner by invoking a series of predefined actions. In a similar study [24], the authors presented a health and emergency care platform for elderly and disabled people to monitor their health status autonomously with a relevant response. In this approach, data are collected from diverse sensors deployed in surroundings and analyzed, and a corresponding action is triggered based on the context determined through fuzzy rules. Researchers also developed a platform as a service [26] whereby people can develop applications in a smart city environment for disabled or older people. The platform has a provision to query and discover relevant services (as a service list) to be utilized in the application. The services are discoverable based on the service description, annotations, and analyzed data. The authors of [27] developed a system in an IoT and smart city context for improved utilization and management of parking spaces allocated for use by people with disabilities. In the education sector, a middleware system was proposed in [28] for the inclusion of disabled people to provide equal education opportunities. The proposed system is called ONTODAPS (ontology-driven disability-aware personalized e-learning system). Education information is collected from a typical education environment, e.g., a university or learning institute and represented in a form compatible with the user disability. ONTODAPS also caters to the needs of users with multiple disabilities through personalization. With the help of a personalization agent, the whole process is automated, adjusting to the needs of the learner. The context is determined based on the student profile information maintained in the database. Most of the above-mentioned techniques utilize workflow-based, rule-based, or context-based predefined service selection to achieve the desired objective in diverse domains.

### 2.3. Quality of Service Factors

Service selection during the composition process is mainly driven by functional quality of service (QoS) criteria, such as efficiency and reliability [29,30,31], scalability [23,32], availability [33,34], and response time [35,36]. In [29,30], the authors did not focus only on the efficient delivery of the services or information but also on the trustworthiness and reliability of data from different sources. In contrast, the authors of [31] focused on information reusability, extensibility, and interoperability. In [32], the authors proposed a framework that allows for dynamic composition of services based on user preferences and device characteristics. The framework is deployed near clients to transform content based on user requirements defined in their one or more profiles, of which performance and scalability are the key concerns, along with privacy protection. In the context of IoT, the authors of [23] developed an efficient and scalable solution to provide collaboration and composition of services among physical objects in the real world. The efficiency of the proposed system is achieved through an existing efficient algorithm, i.e., a graph plan, for automated planning toward service composition, whereas the possibility of extending the current system with additional services makes it scalable.

The availability of required services is one of the fundamental challenges in autonomous or semi-autonomous service-providing systems, i.e., services either do not exist or are difficult to discover in different scenarios. In [33], the authors presented a solution to this problem by abstracting service conversations with clients and a suitable service-combining strategy. Services are grouped using community ontology with semantically similar functionality, whereby the behavior of those services is described in terms of a finite transition system. In another study [34], the authors presented a framework that supports the availability of heterogeneous services developed in various service-oriented architectures (SOAs). A case study in a smart home environment was conducted to evaluate the feasibility and performance of automated service composition compared with the manual process, and it was limited to OSGi- and SOA-based services. In some situations, it is essential to respond quickly to a client’s requests for services. For instance, the authors of [35] developed an efficient algorithm for service selection and composition in the context of robots. They also proposed a minimal-cost formula for optimal dynamic selection of a set of services. A similar study [36] discussed the possibilities of designing an ambient assisted living solution for elderly people, whereby the immediate response to an emergency, i.e., calling for help through a public telephone line, is among the top priority criteria. From our perspective, the inclusion of accessibility QoS properties in the selection process would produce universal augmented services that meet the functional and social demands of people with special needs.

### 2.4. Service Selection Models and Major Challenges

The rapid development in the fields of Internet of Things and pervasive computing has created an abundance of cloud services spanning multiple domains, leading to unprecedented competition between service providers to accommodate the various needs and contexts of users. However, the endeavor of selecting the most satisfying service providers or best services is a complex task, especially in view of the dynamic requirements of users and the presence of many options from more than one service provider. Several studies discussed intelligent service selection methods to choose the best services for a user depending on her preferences, characteristics, current context, rating, and reputation of service providers. 

The service execution engine organizes and supervises the service composition workflow. Each service composition comprises numerous services that can be managed, replaced, and updated in real time without disrupting the business processes that affect QoS attributes. Quality of service (QoS) refers to the integrated online services that make up the service composition, which can be influenced by a variety of internal and external elements, such as the hosting environment, network, and service upgrading [37]. Below, we review several prominent service selection algorithms used during service composition.

The authors of [38] presented an approach based on empirical knowledge that uses the genetic algorithm (EK-GA). This method has a high level of availability, dependability, and scalability. Additionally, it has the potential to reduce energy usage, as well as execution and response times, although with significant latency. The authors of [39] proposed a service composition selection method using the extended Gale—Shapley (GS) algorithm, which generates many service composition options. This approach considers requests with a variety of limitations. Despite a high success rate, low cost and energy consumption, increased reliability, and good scalability, this method has low availability and a long reaction time. In addition, in [40], researchers developed a new chaos control optimal algorithm (CCOA) to address service composition selection problems. The results of the experiments show that the proposed strategy can search for and discover better solutions in less time. Although this method has a short search time, high search quality, high reliability, low energy consumption, and low cost, it has limited scalability.

Moreover, the authors of [41] proposed a novel model based on mixed-integer programming (MIP) that tackles the service composition selection issues using a local search algorithm called HICA. This method provides high quality at low cost; however, it results in low reliability and low scalability. In addition, in [42] researchers proposed a novel solution based on the teaching–learning optimization algorithm to address the service composition selection issues, taking service correlations into account. This strategy has a high level of feasibility, stability, and repute but a low level of accuracy. The authors of [43] proposed a strategy for service composition based on a synergistic elementary service group. To overcome the high computing complexity, researchers deployed a matrix-coded genetic algorithm. Although the solution showed a high success rate, low latency, high reliability, and scalability, it has a high response time.

In addition, the authors of [44] proposed a dynamically selected candidate composite service model based on a cross-modified artificial bee colony algorithm. This approach outperforms other algorithms in terms of response time and accuracy, but the fundamental flaw of this strategy is that it does not include serial task node optimization while assessing the service composition methodology. Furthermore, in [45], researchers used a multi-objective water drop algorithm to handle the problem of selecting a service composition. Although this technology achieves low energy usage, low cost, and low service latency, these benefits come at the cost of scalability. Moreover, the authors of [46] developed a novel hybrid technique for dealing with multi-objective service composition selection based on the ABC algorithm. Although the proposed solution enhances scalability, reliability, and affordability, it has significant latency and complexity. The authors of [47] used a metaheuristic genetic algorithm to handle the problem of service composition selection. The proposed method has a short execution time and a low cost but limited scalability and reliability.

Although several methods have been proposed to enable automatic selection of services, there is a lack of dynamic approaches that deal with the context of users, time of interaction, and service providers. Moreover, we could not find a robust service selection approach to satisfy the needs of disabled users. Hence, the present research provides a framework for an efficient machine-learning-based solution with consideration of people with disabilities and quality of service factors. Our proposed approach pays special attention to a multitude of characteristics specific to disabled users, including their disability profile, preferences, environment, and available IT resources. Moreover, our approach is comprehensive in that not only an inclusive disability ontology for assistive service selection is presented but also a machine learning (ML) framework to choose services adaptively to suit the dynamic requirements of people with special needs. Table 1 presents a comparison of the main service selection approaches and also highlights the contribution of our proposed approach. 

**Table 1:** Summary of prominent service selection models and their weaknesses.

Service Selection Model/Algorithm	Dataset	Accessibility Features	Strengths	Weaknesses
EK-GA [38]	Collected	No	Cost Execution time Response time Availability Reliability Energy consumption	Significant latency
Gale–Shapley algorithm (GSA) [39]	Random	Yes	Cost Execution time Reliability Energy consumption	Low availability, Long reaction time
Chaos control optimal algorithm (CCOA) [40]	N/A	No	Cost Energy consumption Execution time Reliability	Limited scalability
HICA [41]	Random	Yes	Cost Execution time Quality	Low scalability, Low reliability
Hybrid teaching–learning-based optimization (TLO) [42]	Random	Yes	Cost Reputation Reliability Execution time	Poor accuracy
Genetic algorithm (GA) [43]	Random	Yes	Cost Scalability Reliability	Long response time
Cross-modified artificial bee colony (CMABC) [44]	Random	Yes	Execution time Reliability Availability Scalability	Low scalability, High complexity, High energy
Intelligent water droplet (IWD) [45]	Random	Yes	Energy Consumption Cost Latency	Low scalability
ABC [46]	Random	Yes	Cost Availability Execution time Reliability Reputation	Latency, Low scalability
Metaheuristic GA [47]	Random	Yes	Cost Execution time	Low scalability, Low reliability
Our proposed model	Systematically generated	Yes	Response time Availability Accessibility Latency Cost QoS mean QoS rating Accessibility rating	Lack of testing in real situations

## 3. A Motivating Scenario for Accessible Service Selection

We were inspired by the scenario-based design technique suggested in [48] to guide the implementation of our ML-based approach. In scenario-based design, system usage is described to capture the possible user interaction, tasks, and activities in a concrete manner. The key ingredients of good scenarios typically include actors, motivations, capabilities, objects, sets of tasks, and context of use. It is also important to state that user obstacles and challenges are considered in these types of scenarios. The scenario-based design approach empowers us to envisage the possible user interaction and experience and thus infer the correct requirements for our system. Moreover, sketching scenarios that reflect realistic situations is a powerful technique to help developers clarify requirements and make design decisions quickly and efficiently [49,50].

We applied these research recommendations during the development of our motivating scenario [51]. Scenarios must model typical users and their daily activities; scenarios must be abstracted from technical terms and understood by ordinary people; scenarios ought to be centered around users and their interaction rather than system features; and scenarios must be contextualized. Our motivating scenario is applied to the domain of distance learning, where courses are offered online. The scenario is crafted to exhibit variability in user activities, types of user impairments, assistive technologies, services required, service providers, and environment of use. Three users are envisaged in the scenario and share the same goal of obtaining a bachelor’s degree in Islamic studies. Before we delve into the scenario, several assumptions must be highlighted.

- All typical users (in this case, students) in the scenario have some form of disability;
- User demographics, disabilities, characteristics, and goals are collected and stored within dedicated user profiles;
- Users have access to high-speed Internet (e.g., WLAN, WIFI, 4G LTE) during their lectures;
- Users are equipped with smart mobile devices and assistive technologies (AT) to access compatible services; examples of these technologies include smart TVs, smart watches, etc.;
- Location services are enabled on user devices to detect their exact location at the time of online courses;
- Service selection must consider the current user environment and preferences (i.e., context-aware).

The Islamic University of Madinah (Saudi Arabia) recently launched an entirely online bachelor’s degree of art in Islamic history and civilization. The undergraduate degree is taught in the Arabic language and is aimed at students from all parts of the world. What makes the degree unique is its extensive support for students with diverse disabilities to achieve the strategic goals of accessibility, inclusion, and disability-friendly online degrees. The study program employs modern smart technologies to deliver educational materials in different formats (text, audio, videos, etc.) and enable interactive learning as per the needs of disabled students. Students are empowered to connect using video conferencing software, such as Blackboard Collaborate, Microsoft Teams, and Google Meet.

Adam, 25, a deaf student, is enrolled in the degree, as he is fascinated by historical events and lessons. Adam is well acquainted with American Sign Language (ASL) and is highly motivated to tinker with new technologies. However, he tends to forget important deadlines and requires constant reminders about his lectures and assignments. To overcome this problem, he capitalizes on assistive technologies to help him manage his learning activities.

While reading the five things to know this morning news from his living room in Venice (Italy), Adam receives a reminder on his Smart TV 15 min before his live class on the history of peace. As soon as the lesson starts broadcasting from Madinah on his TV, a smart avatar pops up on the screen next to the slides and starts interpreting the Arabic audio into American sign language using artificial intelligence. Adam loves to interact during class with intriguing questions by typing them into a dedicated app installed on his smart TV. The questions instantly appear on the screen of the course instructor, who does not hesitate to start an interesting discussion.

Jenny, 22, a wheelchair user from London (UK), and Ahmed, 30, a blind person from Madinah (KSA), are enrolled in the same degree as Adam. Jenny is keen to learn the Arabic language, but her reading level is currently elementary. At the time of the history of peace lesson, Jenny happens to be at the library, where she enjoys spending her time. She decides to activate the subtitles on her iPad to avoid disturbing fellow students in the library. She also chooses to utilize the translation service to read English subtitles.

Unlike Jenny, Ahmed’s first language is Arabic, so the instruction medium does not pose a challenge for him. However, Ahmed finds it difficult to focus on his online classes with children around him at home; therefore, he frequently visits the nearest blind charity, which provides a quiet learning atmosphere equipped with state-of-the-art learning technologies. Ahmed takes his seat in one of the high-tech learning booths. Amazon Alexa Echo, the voice assistant, connects Ahmed to his online class and enables him to interact with the instructor and request details about his daily schedule.

**Figure 1:**
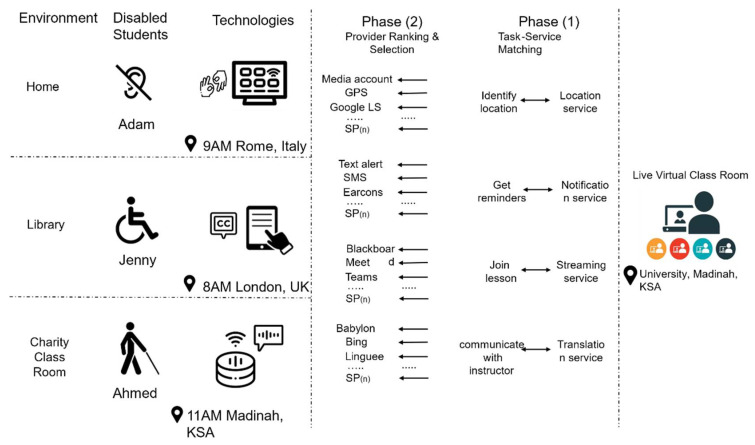
Our working scenario of online learning service selection for students with disabilities. We applied scenario-based design to tackle our research challenge of assistive service selection for disabled people.

To facilitate understanding of the above online learning scenario, Figure 1 depicts a concise overview of the specifics of the users, interactions, and context of the hypothetical scenario.

## 4. An Accessibility Ontology for Service Selection

The authors of various studies have attempted to develop ontologies that describe the accessibility aspects of IT solutions, such as e-learning systems [52]. In this section, we highlight the notable works in this area and argue for the need for our ontology. In short, an ontology refers to the science of entities, the categories of objects, and their dependencies [53]. Domain ontologies provide several advantages [54,55], including the representation of domain knowledge, use of shared vocabulary, reuse and classification of concepts, and linking of entities and their types. With respect to our research, the application of ontologies to guide service selection and composition is not a new prospect [56,57]. Domain ontology concepts are usually used to describe the classification of web services, their inputs and outputs, and their operations. Moreover, ontologies may be used to describe quality of service (QoS) constraints [58]. Prominent languages and tools for creating and managing ontologies include Web Ontology Language (OWL1 and OWL2) [59], Protégé [60], and WebProtégé [61]. A detailed comparison of the features, interaction techniques, and strengths and weaknesses of each ontology development tool is presented in [62,63,64].

For service selection or service composition, the literature provides a few handy ontologies for service selection or service composition. However, none of them are tailored for people with special needs. A recent survey and an in-depth analysis of existing disability ontologies are presented in [65]. A close look at the existing ontologies reveals that none of these ontologies were developed to accommodate all needs of people with disabilities within the topic of service selection. In this research, we review the most prominent accessibility ontologies and devise an appropriate semantic ontology to guide the selection of accessible services as per the context and requirements of people with special needs. The selected concepts must satisfy the following constraints:

- The ontology classes help achieve the specifics of the scenario detailed in Section 3; and
- The concepts of disability ontology help model online education services.

**Figure 2:**
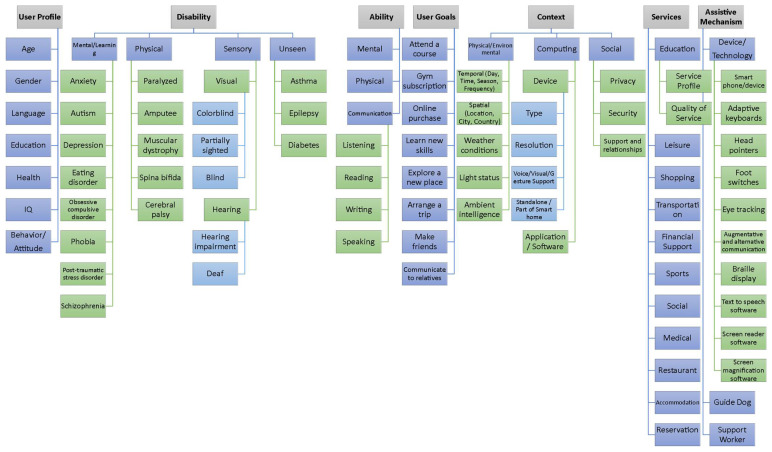
Our proposed disability ontology for enabling assistive service selection. The ontology includes seven main branches, namely user profile, disability types, abilities, user goals, content of use, services, and assistive mechanisms.

Figure 2 presents our comprehensive ontology, which amalgamates most of the relevant service selection concepts to empower disabled people to capitalize on the advantages of augmented services. We derived the disability ontology based on various studies in the literature (Table 2). Most ontologies that were previously proposed in the literature focus on a few aspects of disability. However, our proposed ontology covers many crucial classes that could facilitate the automatic selection of services for disabled people. 

Our proposed ontology considers a multitude of elements that either describe the disabled person’s profile and context or lay down the services that would be useful for accomplishing the required tasks. Additionally, the ontology enables rule building, which can facilitate assistive service selection. The following are the main branches of our proposed disability ontology:

1. User profile;
2. Disability;
3. Ability;
4. User goals;
5. Context;
6. Services;
7. Assistive mechanisms

The user profile is arguably the most important part of the ontology, as the recommendations are typically generated based on the characteristics of our users. A complete user profile can help us make an accurate service selection and therefore achieve improved technology inclusion. We propose to add essential characteristics that have been proven to provide a better selection for disabled persons. These characteristics are age, gender, language, education, health, IQ, and behavior/attitude [72,73,74]. 

The second branch of the ontology includes the characteristics of the disability. We covered most aspects related to a disability, including mental/learning disability, physical disability, unseen disability [75], and sensory disability [74,76]. 

Ability is yet another important branch of our proposed ontology that covers aspects such as mental, physical, and communication abilities. Certain disabilities, such as autism, make it very difficult for a person to process and use or retain useful information [77]. 

User goals can help to identify the short-term and long-term interests and aspirations of disabled persons. By adding user goals to our ontology, we captured crucial tasks, such as attending a course, obtaining a gym subscription, making an online purchase, learning new skills, etc. [67,70,78]. 

Context awareness can help identify and elaborate the needs and preferences of disabled persons. This, in turn, can derive the inclusion of the most relevant service selection. Our proposed ontology includes a wide range of aspects of the disabled person’s context, such as physical/environmental, computing, and social, as suggested in various studies [67,79,80,81,82]. 

Our proposed services branch combines several useful services that can help the disabled person perform daily routine tasks. It includes education, leisure, shopping, transportation, financial support, sports, social, medical, restaurant, accommodation, and reservation [23,70,83,84].

The fast-paced development of technology and easy access to various applications and devices have proven to be supportive for disabled persons. Many tasks can be carried out with the aid of assistive mechanisms, including technology- and non-technology-related mechanisms. Our proposed ontology includes aspects such as device/technology, guide dog, and support worker. The device and technology branch also includes smart phone/device, adaptive keyboards, head pointers, foot switches, eye tracking, augmentative and alternative communication, braille display, text-to-speech software, screen reader software, and screen magnification software [71,75,85,86,87].

## 5. Assistive Services Datasets and Proposed ML-Driven Selection Framework

### 5.1. Generation of Accessible Services Datasets

Because we could not find any ready-to-use accessible service datasets, we had to obtain published services datasets and extend them with scenario-related accessibility features while also considering some of the concepts introduced in our disability ontology, as presented in Figure 2. The outcomes of our data generation exercise were semi-synthetic service datasets, which acted as testbeds for our ML service selection framework. Therefore, the first crucial activity towards implementing and testing our ML-inspired approach was to find web services datasets that fit the objectives of our research. The choice of the datasets had to satisfy several criteria, namely that the datasets (1) are open source for extendibility, (2) contain sufficient real web services, (3) have a clear description of service properties (QoS), and (4) are credible (i.e., have been used by other researchers). 

Our exploration led us to two popular web service datasets. The first dataset includes a total of 339 distinct users, and the second dataset contains about 2507 real web services (referred to as the QWS V2.0 dataset). Further details of these two famous datasets are published in [88,89] and [90,91], respectively. In the following sections, we will describe how we semi-synthetically generated the datasets to incorporate accessibility characteristics related to disabled students and assistive services.

The first dataset contained general information about real users. However, we had to make considerable modifications to the feature variables as per our disabled student scenario. Therefore, we needed to add the form of disability, current place, current time, used tool or device, and first language, in addition to the user tasks required to fulfill the common user goal (attending a live class on the history of peace). The user tasks (i.e., T0 to T11) for this dataset are a set of columns that represent all likely subtasks contributing to the fulfilment of the main goal of the disabled user. Each supported task is assigned a value = 1, whereas unsupported tasks are assigned a value = 0, depending on user profile, type of disability, etc. 

Eventually, we created a dataset containing 1000 disabled users, wherein each user has unique characteristics. The disabled user’s dataset comprised 7 input features (UserID, CommunicationLanguage, DisabilityType, Location, Time, UserDevice, and UserGoal) and 12 output features, as listed in Table 3. The output features correspond to the 12 tasks that are directly linked to the goal of attending online courses, as described in the above scenario, as follows:

- T0: identify user location;
- T1: notify user about class;
- T2: live stream the classroom;
- T3: speech to text (STT);
- T4: text to speech (TTS);
- T5: speech to sign language (STSL);
- T6: sign language to speech (SLTS);
- T7: show subtitles or captions on screen;
- T8: translate text from Arabic to English;
- T9: customize screen color contrast (for visually impaired/low-vision users);
- T10: image text reader (i.e., alternative image text to describe images);
- T11: speech commands (SC).

**Figure 3:**
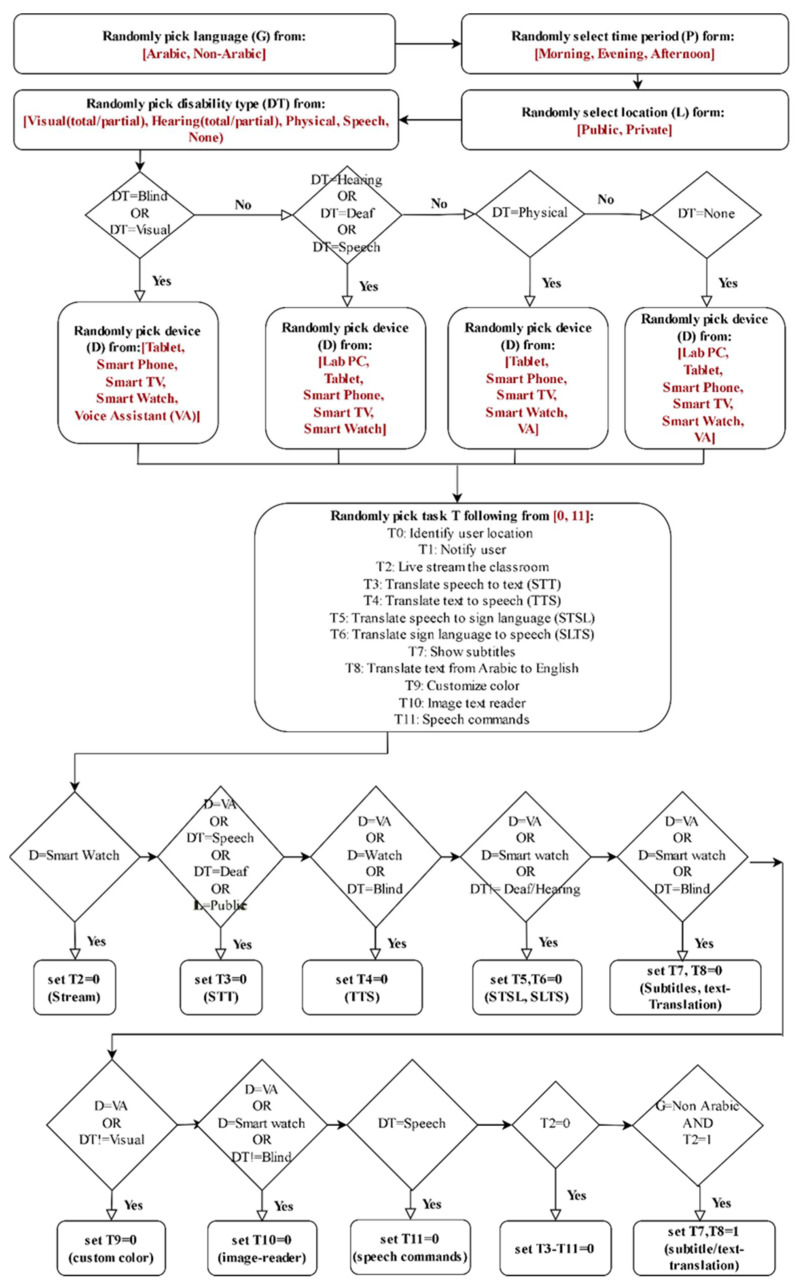
Heuristics for generating the first dataset. The dataset contains 1000 disabled users with differing attributes and 12 user tasks. T = tasks, DT = disability type, D = service, G = language, P = time period, L = location.

To generate our first disabled user dataset, we formulated a heuristic approach, which is governed by predefined rules, as depicted in the flow chart presented in Figure 3. Users and their characteristics are generated in a random manner; however, using rules would produce realistic instances and situations. One such confining rule is as follows.

“If Device is ‘Voice Assistant’ or Disability is not ‘Visual’ then Customize-color is set to 0, as only visually impaired users require the service, and it is not available for Voice Assistant device. Similarly, If Device is ‘Voice Assistant’ or ‘Smart Watch’ or Disability is not ‘Blindness’ then Image-reader is set to 0. Because users with speech disability cannot use the Speech-commands service, it is set to 0 for users with ‘Speech’ disability”.

These heuristics ensure the creation of users with different disabilities and conditions while eliminating unlikely circumstances (e.g., a blind person using a sign language service).

The second dataset encompasses details of the competing service providers that aim to accomplish the 12 subtasks stipulated in the first dataset. This dataset extends the QWS Dataset Ver 2.0. Again, we had to make a few adjustments to fit the dataset to our working scenario by adding accessibility-specific features, as well as relevant services. For further insights about the important QoS metrics to be considered in service selection, see [92].

We ended up creating a service dataset containing 15,000 instances, where each instance exhibits the user rating of a specific service provider that would fulfill an atomic subtask for the online learning scenario. Each record includes the specific QoS of the service provider. Table 4 lists the full feature variables of the second dataset after modification, including user_id, task_id, task, service_name, time, location, response_time, availability, successability, latency, cost, QoSmean, QoS_rating, and Accessibility_rating. 

The dataset covers 12 subtasks (aligned with the first dataset), where each subtask is fulfilled by five possible service providers. The tasks and corresponding services are summarized in Table 5. Each service provider is evaluated randomly by 250 unique users (from the first generated dataset). Therefore, each task received 1250 (250 × 5) user ratings. This gives us (1250 evaluations × 12 tasks), for a total of 15,000 instances.

After selecting the disabled users, the following operations are performed to generate the records for each user rating (i.e., for each service for each task):

1. Time is randomly selected from [‘Morning’, ‘Afternoon’, ‘Evening’];
2. Location is randomly selected from [‘Public’, ‘Private’];
3. QoS metrics are extracted from a randomly chosen row corresponding to a unique service from the QWSDATA Ver2.0 dataset. The following four QoS metrics are selected: response time, availability, successability, and latency;
4. For each of the four QoS metric values extracted from the file, a randomly selected percentage (0–100%) of the same metric value is randomly added to or subtracted from the original value to obtain the new, modified metric value for the dataset;
5. The QoSMean value is obtained by averaging the modified metric values for response time, availability, successability, and latency while inverting the sign for response time and latency;
6. The cost value is randomly selected from a uniform random distribution between 0 and 1.

Finally, the following operations are performed on the complete dataset:

- The QoSMean field is normalized across the complete dataset to a range of 0–1;
- The QoSRating for each entry in the dataset is obtained by averaging the QoSMean with the ‘cost’ and then converting the result to a categorical integer value between 1 and 5 (by integer sampling after multiplying by 5 and adding 1);
- The AccessibilityRating for each dataset entry is obtained by weighted averaging of the QoSMean with a random rating value sampled from a continuous uniform distribution between 0 and 1. The weights for weighted averaging can be set manually and were selected as 2 and 1 for random-value and QoSMean, respectively, in the current dataset.

It is worth noting that some features required label encoding (e.g., categorical values into numeric values) and scaling (i.e., standardization/normalization) into ratios [0, 1] using the below formulas. This helped us to unify the dataset (e.g., the ranges) and eliminate variability across the features, especially when varying scales exist.

(1) $$X\;\left(stand\right)=\frac{Xi-Mean\left(X\right)}{Standard\;Deviation\;\left(X\right)}$$

(2) $$X\;\left(norm\right)=\frac{Xi-Min\left(X\right)}{Max\left(X\right)-Min\left(X\right)}$$

Both of our generated datasets are deposited on GitHub for reuse by the research community [93].

### 5.2. Machine-Learning-Driven Service Selection Framework

**Figure 4:**
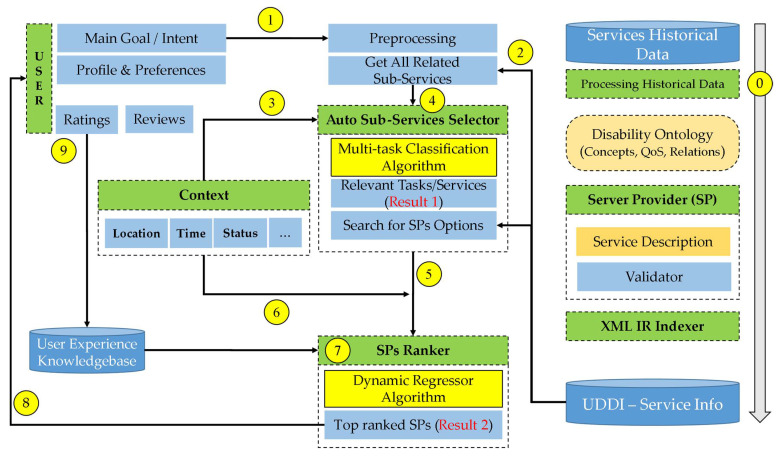
The proposed ML-driven service selection framework for people with special needs. Key components include the disability ontology, validator, UDDI, selector, ranker, and reviews knowledgebase.

The proposed ML-driven service composition framework for selecting assistive services that suit the demands of people with disabilities is depicted in Figure 4. The framework incorporates the basic components and steps (signified via numbers) that are executed within the platform, as well as the necessary processing applied to achieve the desired outcomes. Below, we delve into the working aspects of each component of the proposed framework.

The ontology component represents a summarized classification of the characteristics and factors that could impact people with special needs and their interaction with web and IoT services. The disability ontology also extends to describing user goals and tasks to achieve their endeavors. These tasks can be easily associated with the available services that supply (i.e., by competing service providers) the business logic required to fulfill user tasks while considering the quality factors (QoS) and business offerings of each service. This ontology plays a key role in unifying the mechanism of representation and description of the individual services and their specifics (e.g., input, operations, and outputs) in a way that enables seamless integration of heterogeneous services (e.g., interoperability) from different service providers based on user goals. Thanks to the ontology, the newly composed services are more adaptive to the user demands and needs. Typically, each service provider should register and annotate a new service using the ontology concepts that are supported by the designed service. The ontology has the advantage of determining the candidate tasks associated with a specific service as per the user requests.

When registering a new service to the repository, the validator component checks and validates the service description using standard notation languages, such as XML or JSON. The validator ensures that the service is annotated correctly using the approved ontology concepts.

After adding the description and URI of the new service to the universal description, discovery and integration (UDDI) registry, the indexer indexes the service based on its name, description, and XML information retrieval techniques to facilitate quick and easy connection to all similar services provided by various service providers.

When a composite service is requested by a disabled user (e.g., to fulfill a user intent), the selector component refers to the disability ontology to identify the subtasks that could help to achieve the user goal. Next, the selector fetches the user characteristics (e.g., type of disability and interaction device type) and user context information (e.g., time and place). The selector forwards the user-specific information to our ML model, which is presumably trained on historical data of previous service requests. The ML classifier predicts the appropriateness of the available tasks for the required composite service (i.e., user goal), the nature of the disability, and the user’s current context.

After classifying the subtasks that must be provided to accomplish the user goal, the ranker component ranks and selects the best service provider to fulfill each task, considering user preferences, current context, and previous user evaluations of the service provider with respect to the quality features of service (i.e., QoS). Initially, the ranker identifies all available service providers for each task with the help of an indexer and then applies a machine learning model to predict the ranking of the related service providers. The ranking assigns a degree of correlation to each provider while considering the model’s input (e.g., user preferences, current context, QoS, and previous service ratings). It then ranks the available service providers from best to worst according to the correlation degree to help the framework choose the best service provider. This process is repeated for all tasks that were selected (by the selector component) for inclusion in the composite service.

Finally, the framework closes the loop with a feedback process, which prompts the disabled user to evaluate the service providers of a given task, thus contributing to a knowledge base of previous user experiences. Such user feedback enables the continuous monitoring and tracking of the quality of tasks, services, and service providers.

The details of the machine learning algorithms that were used to guide the decisions of the selector and ranker components are described in Section 5.3.

### 5.3. Multi-Task Classification and Service Provider Matchmaking Algorithms

Apart from the crucial contrast between supervised classifiers and their advantages and disadvantages, our hypothesis in this paper is that choosing the ideal classifier depends on the accuracy of the classification (acc), is computed using a training runtime benchmarking competition method. We considered a small-scale collection of distinct classifiers, including SVM, KNN, NB, DT, and LogR. Each classifier algorithm is implemented using the winner-takes-all strategy [94] for predicting multi-label outputs, where each label represents a binary classification.

Let D be a multi-task dataset, such that tasks (T) are offered by a set of service providers (*S* = {*s*_1_, …, *s_k_*}, *k* ∈ N). We train/validate a set of classical classifiers (*C* = {*c*_1_, …, *c_j_*}, *j* ∈ N on D) with the objective of identifying the best classifiers (*C*^∗^) at training time, as defined in Formula (3).

*C*^∗^ = argmax {train(c*_i_*, D)} (3)

Here, the classifier with highest training accuracy (i.e., *C*^∗^) is employed to predict tasks (Ti) for given user inputs. Likewise, in the second phase, we identify the best regressor model (*R*^∗^; i.e., has height training accuracy) to rank S, the set of available service providers. In Algorithms 1–4, we describe the entire training–validation process to obtain both *C*^∗^ and *R*^∗^. 

**Figure 5:**
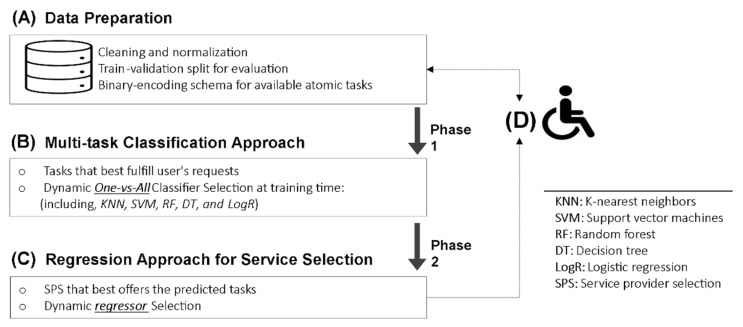
The proposed two-phase service selection approach for finding assistive services. Key phases include (1) multi-label task classification and (2) service provider-to-task matchmaking using a regressor.

Figure 5 summarizes the proposed two phase service selection approach that can help the disabled users find assistive services. The important processes included in this approach are data preparation, multi-task classification approach and regression approach for the service selection. 

**Algorithm 1:** Training procedure to select the best accuracy classifier, *C*^∗^.**Input:***D* = [*I*, *T*]: a training dataset with *I* instances and *T* tasks (labeled tasks obtained
      from our ontology model,
      containing attributes, e.g., UID, Goal, Language, Country, Current Place, Current
      Time, User Device,
      Disability Type), such that each *t_i_* ∈ [0, 1].
      {*C^i^*, *i* = *1*, …, *j*}: a set of classification algorithms.
**Output:**
*C*^∗^: a multi-task classifier with the highest training accuracy.
*/ / Dataset preprocessing:*
   *D’* ← encode(*D*[0, *I*: ∗, ∗]) *convert all t_s_ tasks values in I independently to a binary value.*
   *D’’* ← standardize(*D’* [∗, ∗: 0, *T*]) *normalize all features using min-max scaling.*
*/ / Training and identifying the best classifier according to Equation (*3*):*
*C*^∗^ ← ∅
**for**
*i* ← *1* **to**
*j*
**do** 
   $C_{acc}^{i}$ ← train(*D’’, C^i^*) *training using one-vs-all method*
   If $C_{acc}^{i}$ > $C_{acc}^{*}$ then $C_{}^{*}$ ← $C_{}^{i}$ 
**return**
C*

**Algorithm 2:** Validation procedure to predict a set of tasks, *T*, using *C*^∗^ classifier for each disabled user.**Input:***C*^∗^: a multi-task classifier with the highest training accuracy, obtained by Algorithm 1.
      *V^I^*^×N^: a validation dataset containing *I* examples with N input features.
      *θ*: a predefined probability threshold with a default setting of *θ* = 0.5.
**Output:**
*O^I^*^×|*T*^^|^: a set of predicted tasks (ontological atomic tasks) for all users, *I.*
*V*
*← preprocessing(V^I^^×N^) / / preprocessing the validation dataset as shown in Algorithm* 1.
*O*
*← C*^∗^*.predict(V)*
**for**
*j* ← *1* **to**
*I*
**do**
   If *O*[*j*,*k*] > *θ* **then** *O*[*j.k*] ← *1*: *O*[*j.k*] ← *0*
**return**
*O*

With practically identical procedures for training/validating the best multi-task classifier (i.e., *C*^∗^, discussed in Algorithms 1 and 2), we train and validate a regressor model, *R*^∗^ (see Algorithms 3 and 4), to estimate the best service provider for a given task. Here, *R*^∗^ attempts to rank all service providers that can perform the requested task Ti (predicted by Algorithm 2), such that Si with the highest ranking would be selected to provide Ti.
**Algorithm 3:** Training procedure to select the best training-accuracy regressor, *R*^∗^.**Input:***D* = [*I*, *S*]: a training dataset with *I* instances and *S* labeled service providers. {*R^i^*, *i* = *1*, …, *j*}: a set of regressor algorithms.
**Output:**
*R*^∗^: a regressor model with the highest training accuracy.
*/ / Dataset preprocessing:*
*D* ← preprocessing(*D*) */ / preprocessing the training dataset almost the same as performed in Algorithm*
*1*
*/ / Training and identifying the best regressor:*
R* ← ∅
**for**
*i* ← *1* **to**
*j*
**do**
$\;R_{acc}^{i}$ ← train (*D,* ***R****^i^*) *training with applying a grid-searching method for optimizing the hyperparameters.*
      **If** $R_{acc}^{i}$ > $R_{acc}^{*}$ then $R_{}^{*}$ ← $R_{}^{i}$
return R*

**Algorithm 4:** Validation procedure to predict the best service provider, *S_i_*, for each task, *T^i^*, using *R*^∗^ model.**Input:***R*^∗^*:* a regressor model with the highest training accuracy, obtained by Algorithm 3.
      *V^T^*^×N^*:* a validation dataset containing T tasks with N input features.
      *θ:* a predefined probability threshold with a default setting of θ = *0.5*.
**Output:**
*O^T^*^×|*S*^^|^: a predicted service provider for each task, T.
*V*
*← preprocessing(V^I^*^×N^*) / / preprocessing the validation dataset as performed in Algorithm* 1.
*O*
*← R*^∗^*.predict(V)*
**for**
*j* ← 1 **to**
*I*
**do**
      **for**
*k* ← 1 **to** |T| **do**
            If *O*[*j*,*k*] < *θ* **then** *O*[*j.k*] ← ∅
**return**
*O*

## 6. Results

In this section, we review the performance criteria that were used to judge the prediction power of our algorithms. We also provide a succinct summary of the experimental results and infer the relevant conclusions.

### 6.1. Performance Evaluation Metrics

We used the standard classification metrics that are well-known to judge the quality of ML model predictions, namely accuracy, precision, recall, F1-score, specificity, false-positive rate (FPR), and ROC [95]. These metrics form the so-called confusion matrix, which offers insights into the correctness of our predictions.

Accuracy represents the ratio of the correct predictions out of all predictions produced by the model, i.e., how many times (the true positives and true negatives) the instances were classified correctly by the model (see Equation (4)). Precision indicates the ratio of the correctly predicted positives out of all positive predictions (whether correct or incorrect) generated by the classifier (see Equation (5)). Precision refers to how well the model predicts the positive cases of a particular class. Recall refers to the ratio of the number of true positives classified by the model out of the total actual positive cases (i.e., the instances that should have been classified as positive (see Equation (6))—also referred to as sensitivity. The F1 score is a harmonic mean metric that aims to balance precision and recall, limiting the effect of class imbalance to achieve a high level of precision (reducing type I errors), without missing significant instances (reducing type II errors) (see Equation (7)). The true negative rate (specificity; see Equation (8)) and false-positive rate (see Equation (9)) refer to ratio of correctly predicted negative instances (type II errors) and incorrectly predicted positive outcomes (type I errors), respectively. The metrics are defined by the following formulas:

(4) $$Accuracy=\frac{True\;Positive+True\;Negative}{True\;Positive+False\;Positive+True\;Negative+False\;Negative}$$

(5) $$Precision=\frac{True\;Positive}{True\;Positive+False\;Positive}$$

(6) $$Recall\;\left(Sensitivity\right)=\frac{True\;Positive}{True\;Positive+False\;Negative}$$

(7) $$F1-Score=2*\;\frac{Recall*Precision}{Recall+Precision}$$

(8) $$True\;Negative\;Rate\;\left(Specificity\right)=\frac{True\;Negative}{True\;Negative+False\;Positive}$$

(9) $$False\;Positive\;Rate=\frac{False\;Positive}{True\;Negative+False\;Positive}$$

where True Positive (TP) refers to the number of instances predicted as positive that are positive in reality, False Positive (FP) refers to the number of instances predicted as positive but that are negative in reality, True Negative (TN) refers to the number of instances predicted as negative that are negative in reality, and False Negative (FN) refers to the number of instances predicted as negative but that are positive in reality.

### 6.2. Benchmark Selection Approaches

After the datasets were generated, as explained in Section 5.1, we started the testing phase of our proposed framework. The framework utilizes two main algorithms (Algorithms 1 and 2) to produce its tasks and service provider predictions. The first algorithm guides the selection process for the tasks related to the user goal in accordance with the context and the nature of the user disability. The second algorithm guides the ranking process of the available service providers for each task, thus enabling the selection of the best service provider for each task. In the second algorithm, ranking is performed based on the predicted overall rating score of all service providers. We split our datasets based on the 80–20 rule for training and testing of our model. The Google Colab platform was used to execute our Python scripts of the proposed algorithms and test their performance on our generated datasets. Google Colab allowed us to calculate the ML performance metrics discussed earlier.

We performed binary classification following the ML approach detailed in Section 5.2 to resolve the multi-labelling issue of all user tasks in the first dataset. The online course tasks for each user in the first dataset are classified into two class instances (0 = irrelevant, 1 = relevant) depending on whether the tasks are appropriate to complete the user goal based on the characteristics of the disabled student. Consequently, all tasks were represented as binary vectors. The multi-task classification approach applies five prominent classification machine learning algorithms (i.e., KNN, SVM, RF, DT, and LogR) to the first dataset. To this end, we applied the one-vs.-all strategy in the binary classification to predict the appropriateness of each task based on the existing features. In the one-vs.-all strategy, the winner classifier takes all, which means that the best class classifier is the classifier with the best accuracy score. Obviously, the best binary classifier could differ from one user to another depending on disabled user characteristics and context.

**Figure 6:**
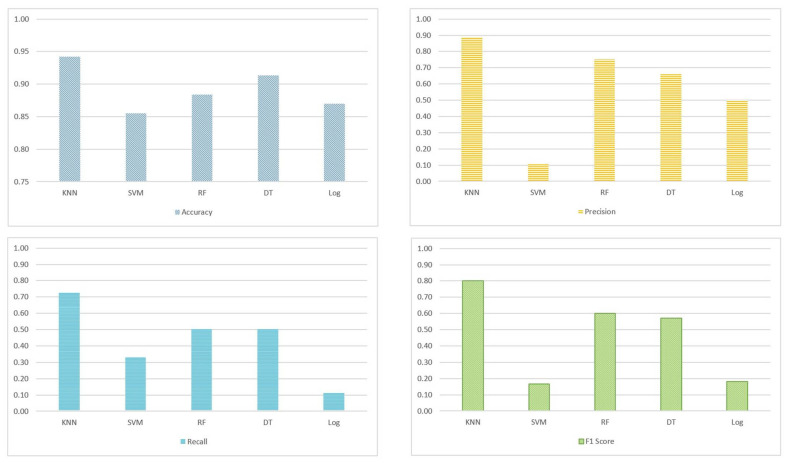
Comparative performance results of the multi-label task selection algorithm (Algorithm 1) using the best classifier (KNN, SVM, RF, DT, and LogR). Analyzed metrics include accuracy, precision, recall, and F1 score.

Figure 6 depicts the test results of the multi-task classification algorithm (using user goal, type of disabilities, and context) and performance of the different ML classifiers (namely KNN, SVM, RF, DF, and LogR). The findings show the superiority of KNN (the winner) with respect to all classification metrics. KNN achieved an accuracy of 94% and an F1 score of 80%. This is considered an acceptable accuracy, given the nature of the first algorithm and the selection threshold (80%) that we used for accepting the online learning tasks. Precision scores are slightly higher than the recall scores; thus type I errors are reduced to a considerable extent. This means that the important tasks required by each disabled student are most likely identified correctly by our model. However, the model may select less important tasks to contribute to the user goal (Type II error). SVM and LogR algorithms achieved the least accurate estimates, especially with respect to accuracy and F1 score.

**Figure 7:**
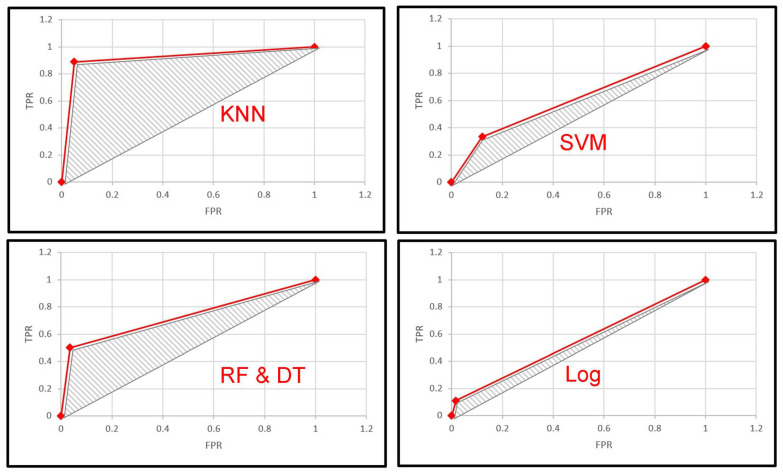
Comparative performance results of the multi-label task selection algorithm (Algorithm 1) using the AUC-ROC metric (KNN, SVM, RF, DT, and LogR).

Figure 7 shows the receiver operating characteristic (ROC) curve for each learning model (i.e., KNN, SVM, RF, DT, and LogReg). In terms of classification, the ROC curve demonstrates the diagnostic power of a binary classifier at different classification thresholds. Essentially, the ROC curve plots the true positive rate (TPR; Equation (7)) and false-positive rate (FPR; Equation (8)). The graphs confirm the superiority of KNN, as it achieved the largest area under the curve (AUC), demonstrating its ability to predict the appropriateness (or lack thereof) of the tasks based on the disabled user profile. Again, LogR and SVM algorithms showed the worst level of separation capacity (i.e., making incorrect predictions about the tasks/services).

Next, we tested the service selection matchmaking algorithm using the second dataset. Our analysis focused on the quality of selection of the best service provider among a set of competing service providers. The selection takes into consideration the quality factors (QoS) of the provider, implicated cost of the service, and prior disabled user ratings.

**Figure 8:**
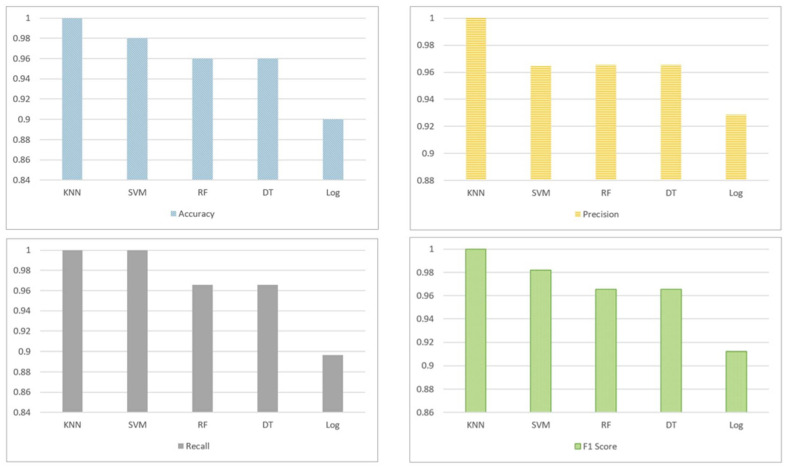
Comparative performance results of the matchmaking algorithm (Algorithm 2) using the one-vs.-all strategy (KNN, SVM, RF, DT, and LogR). Measured metrics include accuracy, precision, recall, and F1 score.

Figure 8 compares the provider–task matchmaking approach in terms of accuracy, precision, recall, and F1 score. The KNN model outperformed other single ML models, achieving 100% across all performance metrics in terms of matching the best service providers to the assistive tasks determined in the first phase (i.e., multi-task classification). LogReg produced the lowest prediction scores among all classifiers.

### 6.3. Comparative Analysis

To extend our evaluation, we compared the proposed two-phase ML-based service selection model with other multi-criteria decision-making models (MCDM). We chose four MCDM models, specifically AHP, SAW, PROMETHEE, and TOPSIS. Each of these models was used to determine the best possible service provider from a set of rival service providers.

When comparing MCDM approaches, the authors of [96] suggest recruiting an expert to inspect the service selection performance. In our experiment, we utilized the same strategy to explore the performance of our approach and existing MCDM models.

**Figure 9:**
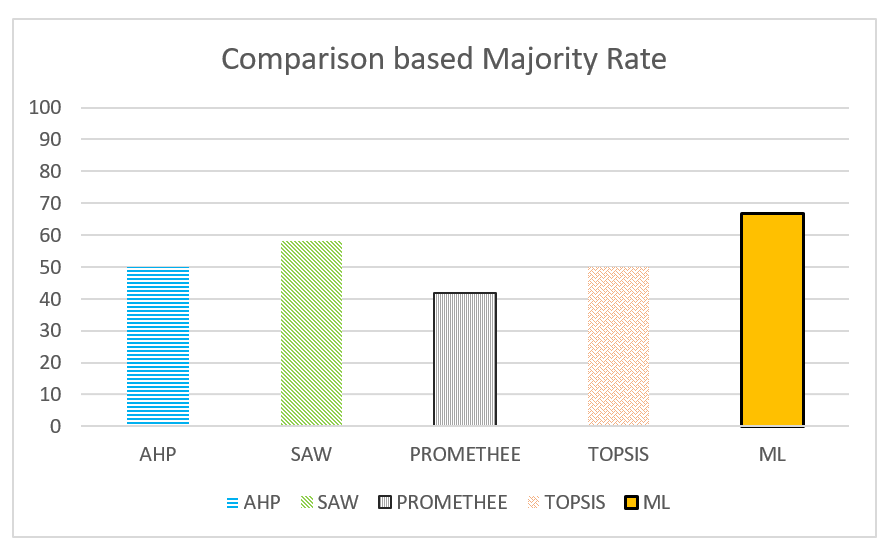
Comparison results of our ML approach against other MCDM models using the majority voting approach. The percentage value represents the agreement with the majority choice. AHP = analytical hierarchy process, SAW = simple additive weighting, PROMETHEE = preference ranking organization method for enrichment evaluation, TOPSIS = technique for order preference by similarity to ideal solution, ML = our ML-driven two-phase approach.

To reduce the human bias in the testing phase, we used the principle of majority voting, also known as the BORDA method [97]. In this approach, several experiments are carried out, and after each experiment, the best option is determined based on the majority choice of all participating or competing models. Thus, after running the experiment 10 times, the majority ratio was calculated, and then the percentage of convergence between each approach with the majority ratio was calculated. Figure 9 shows the superiority of our ML approach over the other MCDM models, with a percentage of agreement with the majority choice of about 68%.

## 7. Discussion

### 7.1. Key Findings

In the era of IoT and cloud computing coupled with the abundance of digital services, with this study, we attempted to tackle the predominant challenge of service selection for people with disabilities. Disabled people constitute a significant portion of the world’s population (e.g., 26% in the USA), and their use of assistive technologies and devices is on the rise, with the aim of helping them complete basic daily functions and live independently. Diverse and evolving user needs/preferences have instigated researchers to develop intelligent methods to select the best services among candidate services. To this end, we proposed a multi-faceted ML approach to recommend the best assistive services that accommodate the requirements of disabled users while considering their characteristics and context.

Existing solutions approach the prospect of service selection from different angles. For example, multi-criteria decision analysis techniques (MCDA/MCDM) are among the popular solutions applied to rank services by assigning weights to quality-of-service attributes, thus guiding the service selection process [7]. MCDA techniques assess, rate, and select from a set of alternatives based on multiple interests/preferences to achieve a general goal. Typically, the alternatives are rated from best to worst using an appropriate scale with respect to satisfying a specific goal. On the other hand, machine learning approaches have gained momentum with respect to selecting the best service providers in various domains, such as cloud computing. For instance, the authors of [98] suggested a random forest regressor to train MCDM models for ranking cloud services. In our research, we opted to compare our ML approach with four prominent MCDA techniques, namely AHP, SAW, TOPSIS, and PROMETHEE.

We propose an intelligent multi-level classification framework that segments the selection task into two phases. In the first phase, the services are determined for each disabled user. In the second phase, service providers for each task are ranked.

The results contribute in four ways. First, they exemplify the successful application of scenario-based design in accomplishing our research objectives. This user-centric approach enabled us to describe the specific tasks and context of assistive services in a lightweight and interactive manner. Second, we propose a detailed ontology for people with special needs to guide the selection process. The ontology covers important classes to address the needs and services of disabled users while considering the types of disability and context of use. Third, we enriched existing services datasets by encompassing accessibility/disability features. The generated datasets can help researchers investigate other research problems, especially those linked to the impact of disabilities on service selection. Forth, we described a two-phase machine learning service selection framework, along with multi-label task classification and service provider regressor algorithms.

Our solution is a software framework that can have real-world implications, as it allows for different genres of services to be integrated and selected, whether software or hardware services. Although no real-life testing was conducted at this stage, in the future, it could be possible to integrate IoT services and devices, such as in [99], into the framework to fulfill the demands of various types of disabilities.

### 7.2. Limitations and Threats to Validity

In this section, we will delve into some limitations that could affect the quality of our findings. One of the critical threats to the external validity of our results is data bias. We were not able find or collect a real service dataset for people with disabilities, which represents a research gap that should be addressed by the research community. Therefore, we resorted to creating synthetic datasets that encompass disability features. We made some assumptions and implemented heuristics to create dataset instances representing disabled users and service providers. However, the datasets incorporate feature variables that are highly specific to the online education scenario. Exploring other application areas, such as smart homes and smart health, would require a significant adjustment to the proposed datasets.

The suggested framework was tested on one scenario using two datasets only. To assess the generalizability of the ML-based framework, validation should be extended to cover a multitude of scenarios and datasets.

The association between the input features (impairment aspects and QoS) and dependent variables (i.e., subtasks and service providers) merits further assessment through, e.g., statistical testing. Moreover, other predictor variables pertaining to disabled users and service providers were not included in the datasets or analysis. Examples of disability features include social and economic factors, whereas examples of QoS factors include bandwidth, privacy, reputation, and customer support.

## 8. Conclusions and Future Works

With this study, we investigated the selection of services tailored for people who suffer from incapacities. Profuse technology services of variable quality are offered by a myriad of service providers. However, selecting the best offerings among those competing services continues to trigger considerable traction in the research community.

Our proposed solution was inspired by the scenario-based design technique, which empowered us to examine the research challenge from a user-centric point of view. As such, we drafted a realistic interaction scenario focusing on the requirements and attributes of disabled users. Next, we synthesized a detailed accessibility ontology that considers past ontologies and captures the specifics of our scenario. The lack of disability service datasets motivated us to create two service datasets that encompass the characteristics of people with disabilities. We invite fellow researchers to use and extend our datasets in their research. The pinnacle of our contribution is represented by a machine-learning-driven framework of assistive service selection. Selection is accomplished in two subsequent steps—subtask classification and service provider matchmaking—to assist in assembling assistive services that support the everyday demands and activities of people with special needs.

This research can be extended by orchestrating smart assistive services for disabled people and collecting relevant features and metrics (preferences, type of disabilities, and QoS) from a real context of interaction. Future extensions would have to integrate other advanced deep learning approaches (CNN, etc.) into our framework to significantly improve the service selection predictions, especially in complex situations and scenarios. In the future, we will implement the full framework as a web application and connect different types of assistive services. Then, we will expose the functionalities of the framework through a dedicated web service made available to disabled users to help them exploit the best services according to their preferences and current context.

## Figures and Tables

**Table 2:** Summary of notable ontologies incorporating disability aspects and their domains of implementation.

Study	Concepts	Domains of Ontology
[66]	Type of disability	Transportation, tourism, and education
[67]	Context (environment, computing, physical, user)	E-learning
[68]	Personal characteristics, such as age, gender, education, income, etc.	Mobility
[69]	Personal preferences and characteristics, such as preferred hotel location, mode of transportation, medical status, etc.	Hotels, transportation; medicine, and cities
[23]	Personal preferences, such as required temperature and humidity level, services, etc.	IoT-based smart home environment
[70]	Personal characteristics, such as interests, goals, and activities Context, such as environment Type of disability	Mobile applications
[71]	Type of disability, communication methods, and medical profile	Health and IoT

**Table 3:** Summary of feature variables of the first disabled users dataset.

Feature Variable	Type	Explanation	Possible Values
User ID	Long	Unique Identifier	1–1000 (number of generated users)
Time	String	The local time of the user when accessing the services	Morning, afternoon, evening
Language	String	The main language that the user can use to communicate fluently	Arabic, non-Arabic
Location	String	The location from which the user is accessing the services	Public, private
Disability type	String	The type of impairment suffered by the user	Blind, visual (Impairment), deaf, hearing (Impairment), speech, physical, none
User device	String	The interactive technology used to access the assistive services	PC, smart TV, tablet, smart phone, smart watch, voice assistant
Tasks (T0 to T11)	Integer	The possible tasks necessary to access the online course	0 (= not supported), 1 (= supported)

**Table 4:** Summary of feature variables of the second service providers dataset.

Feature Variable	Type	Explanation	Possible Values
User ID	Long	Unique Identifier	1–1000 (number of generated users in Dataset 1)
Task ID (0–11)	Integer	The task ID	0–11
Task name	String	The name of the task/service that helps fulfill the online course scenario	Task name from the above list
Service provider name	String	The corresponding service providers for each possible task	Each task is accomplished by five different service providers
Time	String	The local time of the user when accessing the service providers	Morning, afternoon, evening
Location	String	The location from which the user is accessing the services	Public, private
Response	Double	The average time spent to send a request to the service provider and receive a response	Any number of milliseconds
Availability	Double	The number of successful SP invocations (out of total invocations)	Generally varies in the range of 0–100
Successability	Double	The number of successful responses (out of total request messages)	Generally varies in the range of 0–100
Latency	Double	The time spent by the service provider to respond to a specific request	Any number of milliseconds
Cost	Double	The cost of using the service provider	Ranges from 0 to 1
QoSMean	Double	The average QoS of four metrics (response, availability, successability, and latency)	The mean ranges from 0 to 1
QoSRating	Integer	The average user rating of the QoS factors of the service provider	The rating ranges from 1 to 5
Accessibility rating	Integer	The average user rating of the accessibility features of the service provider	The rating ranges from 1 to 5

**Table 5:** Service providers for each online learning task.

Task Name	Competing Service Providers
‘Identify user location’	‘Location_service’, ‘Geo_location’, ‘Online_location’, ‘Live_location’, ‘Location_detection’
‘Notify user about class’	‘Notifications’, ‘Notify’, ‘Push_notifications’, ‘Notify_messages’, ‘Notify_emails’
‘Live stream the classroom’	‘Google_meet’, ‘Zoom’, ‘Microsoft_teams’, ‘Skype’, ‘Slack’
‘Speech to text’	‘Google_ASR’, ‘Microsoft_ASR’, ‘Apple_ASR’, ‘Free_ASR_service’, ‘Online_ASR’
‘Text to speech’	‘Google_TTS’, ‘Microsoft_TTS’, ‘Apple_TTS’, ‘Free_TTS_service’, ‘Online_TTS’
‘Speech to sign language’	‘Sign_language_creation’, ‘Signaling’, ‘Sign_speech’, ‘Gestures_service’, ‘Sign_language’
‘Sign language to speech’	‘Sign_language_interpretation’, ‘Signaling’, ‘Sign_speech’, ‘Gestures_service’, ‘Sign_language’
‘Show subtitles or captions on screen’	‘Subtitles’, ‘Captions’, ‘Transliteration’, ‘Google_STT’, ‘Caption_service’
‘Translate text from Arabic to English’	‘Machine_translation’, ‘Google_translate_API’, ‘Online_translation’, ‘Arabic_translation’, ‘Live_translation’
‘Customize screen color contrast’	Apple_resolution’, ‘Windows_resolution’, ‘color_contrast’, ‘High_res_colors’, ‘Greyscale_conversion’
‘Image text reader’	‘Image_caption’, ‘OCR’, ‘Image_characters’, ‘Image_to_text’, ‘Image_reader’
‘Speech commands’	‘Alexa’, ‘Voice_assistance’, ‘Apple_assistant’, ‘Google_commands’, ‘speech_keywords’

## Data Availability

Our systematically generated disability service datasets are publicly archived at: https://github.com/anamoun/servicesfordisabled.git (accessed on 5 May 2022).
